# Brain Metastatic Prostate Adenocarcinoma: Avoiding Mistaken Identities

**DOI:** 10.7759/cureus.69282

**Published:** 2024-09-12

**Authors:** James A Knight, Andre N Ene, Riham H El Khouli, Zin W Myint, William St Clair

**Affiliations:** 1 Radiation Medicine, University of Kentucky, Lexington, USA; 2 Pathology and Laboratory Medicine, University of Kentucky, Lexington, USA; 3 Radiology, University of Kentucky, Lexington, USA; 4 Internal Medicine, Medical Oncology Division, University of Kentucky, Lexington, USA

**Keywords:** androgen depriving therapy, ga-68 psma (gallium-68 prostate specific membrane antigen), gamma knife (gk) radiosurgery, isolated brain metastasis, oligometastatic prostate cancer

## Abstract

Brain metastatic carcinoma is a rare occurrence among prostate cancer metastases. ^68^Gallium prostate-specific membrane antigen PET-CT ([^68^Ga]PSMA PET/CT) is commonly used for prostate cancer staging and detection of biochemical recurrences. However, various CNS tumors exhibit activity on [^68^Ga]PSMA PET/CT and may often be included in the differential diagnosis. Herein, we present a case of brain metastatic prostate cancer successfully treated with surgical resection and adjuvant stereotactic Gamma Knife radiosurgery (GKRS) followed by androgen deprivation therapy (ADT) to emphasize the need for histologic confirmation. A 70-year-old male with a history of very high-risk prostatic adenocarcinoma presented with biochemical recurrence following radical prostatectomy and irradiation of the prostatic fossa. [^68^Ga]PSMA PET/CT and MRI identified a solitary lesion in the left occipital lobe; differential diagnosis included prostate metastasis, meningioma, or a new metastatic primary lesion. The patient underwent surgical resection, and immunohistochemical staining confirmed the lesion as brain metastatic prostate adenocarcinoma. One month after resection, the patient underwent GKRS to the tumor bed and two additional metastases, followed by ADT. Repeated imaging 15 months after GKRS revealed stable posttreatment changes with no evidence of new metastases, thus demonstrating durable, effective local and systemic control. Brain metastatic prostate adenocarcinoma without nodal or osseous metastases is a rare phenomenon. The affinity of [^68^Ga]PSMA PET/CT for non-prostate histologies such as meningioma introduces uncertainty into the diagnostic process. This case demonstrates the durable local control conferred by GKRS toward these lesions and emphasizes the need for clinical, radiographic, and histopathologic data to identify disease presentations and facilitate appropriate treatment regimens.

## Introduction

Prostate cancer is the most common non-cutaneous malignancy in men [1,2]. Metastasis to the axial skeleton and the lymph nodes is the most typical pattern of spread for prostate cancer [2-4]. Brain metastatic prostate cancer is a rare phenomenon, accounting for 0.2-2.0% of all prostate cancer metastases, and non-adenocarcinoma histologies have been shown to have a higher likelihood of brain metastases than adenocarcinomas [4-7]. Brain metastatic prostate cancer most frequently occurs in the setting of widely disseminated bone and soft tissue disease, making solitary parenchymal metastases without intervening nodal or osseous metastases particularly rare [4]. Here, we present a case of exclusively brain metastatic prostate cancer, identified on ^68^Gallium prostate-specific membrane antigen PET-CT ([^68^Ga]PSMA PET/CT) and successfully managed with surgical resection and adjuvant stereotactic Gamma Knife radiosurgery (GKRS), followed by androgen deprivation therapy (ADT).

## Case presentation

**Figure 1:**
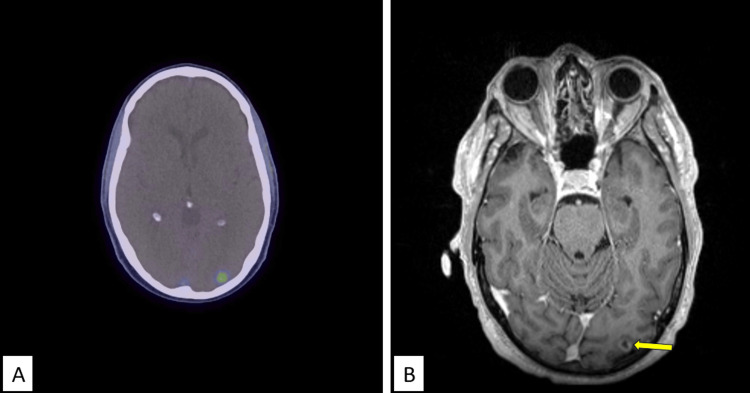
Brain metastatic prostatic adenocarcinoma visualized on [68Ga]PSMA PET/CT and double-contrasted MRI (A) Local PSMA activity fusing to the left occipital lobe peripherally. (B) High-resolution gadolinium double-contrast MRI of the brain revealed a solitary 8.5 mm × 7.0 mm × 9.5 mm peripherally enhancing parenchymal lesion (yellow arrow) with surrounding vasogenic edema. [^68^Ga]PSMA PET/CT, ^68^Gallium prostate-specific membrane antigen PET-CT; PSMA, prostate-specific membrane antigen

A 70-year-old man with an Eastern Cooperative Oncology Group performance status of 0 and a medical history significant for hypertension and hypercholesterolemia initially presented with an elevated pre-treatment prostate-specific antigen (PSA) level of 4.95 ng/mL. Prostate biopsy and subsequent radical prostatectomy yielded a diagnosis of stage IIIC (pT3aN0M0) prostatic adenocarcinoma, with Gleason 4 + 5 = 9 disease. His first postoperative PSA was collected three months after surgery, showing a nadir of 0.13 ng/mL. A subsequent PSA at four months post-surgery returned at 0.19 ng/mL, raising concern for biochemical recurrence. Because the patient’s PSA levels were below the resolution of detection on [^68^Ga]PSMA PET/CT, he was empirically given 66 Gy in 33 fractions of daily salvage external beam radiotherapy targeting the prostatic fossa. Fifteen months after salvage irradiation, [^68^Ga]PSMA PET/CT was performed due to a gradual increase in PSA from 0.27 ng/mL to 1.28 ng/mL over that time period. Imaging revealed focal PSMA activity fusing to the left occipital lobe peripherally with no definite corresponding lesion on the non-contrasted CT images. High-resolution gadolinium double-contrast MRI of the brain revealed a solitary 8.5 mm × 7.0 mm × 9.5 mm peripherally enhancing parenchymal lesion with surrounding vasogenic edema (Figure 1).

**Figure 2:**
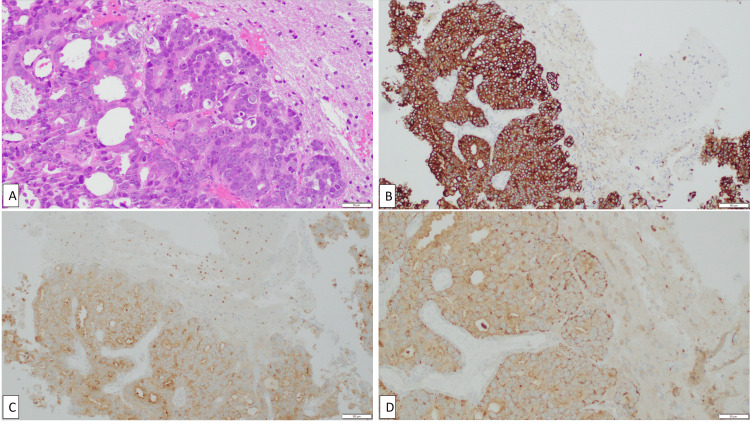
H&E staining and immunohistochemistry of prostate cancer metastases resected from the left occipital lobe (A) Tumor cells on H&E staining at 20X magnification in a cribriform pattern in a background of reactive neural tissue. The tumor cells are pleomorphic, with round to polygonal nuclear contours and open and condensed chromatin patterns with prominent nucleoli. (B) The tumor cells show strong cytoplasmic staining of CK AE1/AE3. Neural tissue is negative for CK AE1/AE3. (C) PSA 10X immunohistochemistry. The tumor cells are decorated by PSA in a cytoplasmic staining pattern; the nuclear PSA staining that would be seen in glial cells is negative. (D) PSAP 20X immunohistochemistry. The tumor cells are decorated by PSAP with cytoplasmic staining. Neural tissue is negative. PSA, prostate-specific acid phosphatase; PSAP, prostate-specific acid phosphatase

The patient underwent left occipital craniotomy and tumor resection. The immunohistochemical profile was strongly positive for cytoplasmic staining of PSA, cytokeratin monoclonal antibodies AE1/AE3, and prostate-specific acid phosphatase (PSAP), but negative for nuclear PSA staining, confirming the tumor as brain metastatic prostate adenocarcinoma (Figure 2).

**Figure 3:**
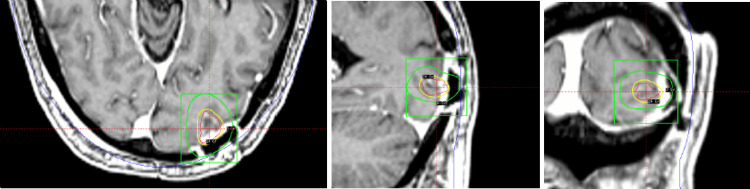
Left occipital tumor bed treated on GKRS (A) Axial, (B) coronal, and (C) sagittal views of the tumor bed, measured at 13 mm × 13 mm × 10 mm and treated to 24 Gy prescribed to the 50% isodose line. GKRS, Gamma Knife radiosurgery

Despite the resection, the patient’s one-month postoperative PSA was found to have risen to 5.42 ng/mL; [^68^Ga]PSMA PET/CT was ordered, revealing interval development of mild PSMA avidity involving two pelvic lymph nodes, a left-sided mesorectal fascia nodule, and the S1 and S2 sacral nerve roots, raising concern for metastatic disease. At one month post-resection, the patient received GKRS to the surgical bed (Figure 3), and two additional sub-centimeter metastases were discovered on a thin-cut MRI with 1 mm slices. Each lesion received a single fraction of 24 Gy prescribed to the 50% isodose line.

**Figure 4:**
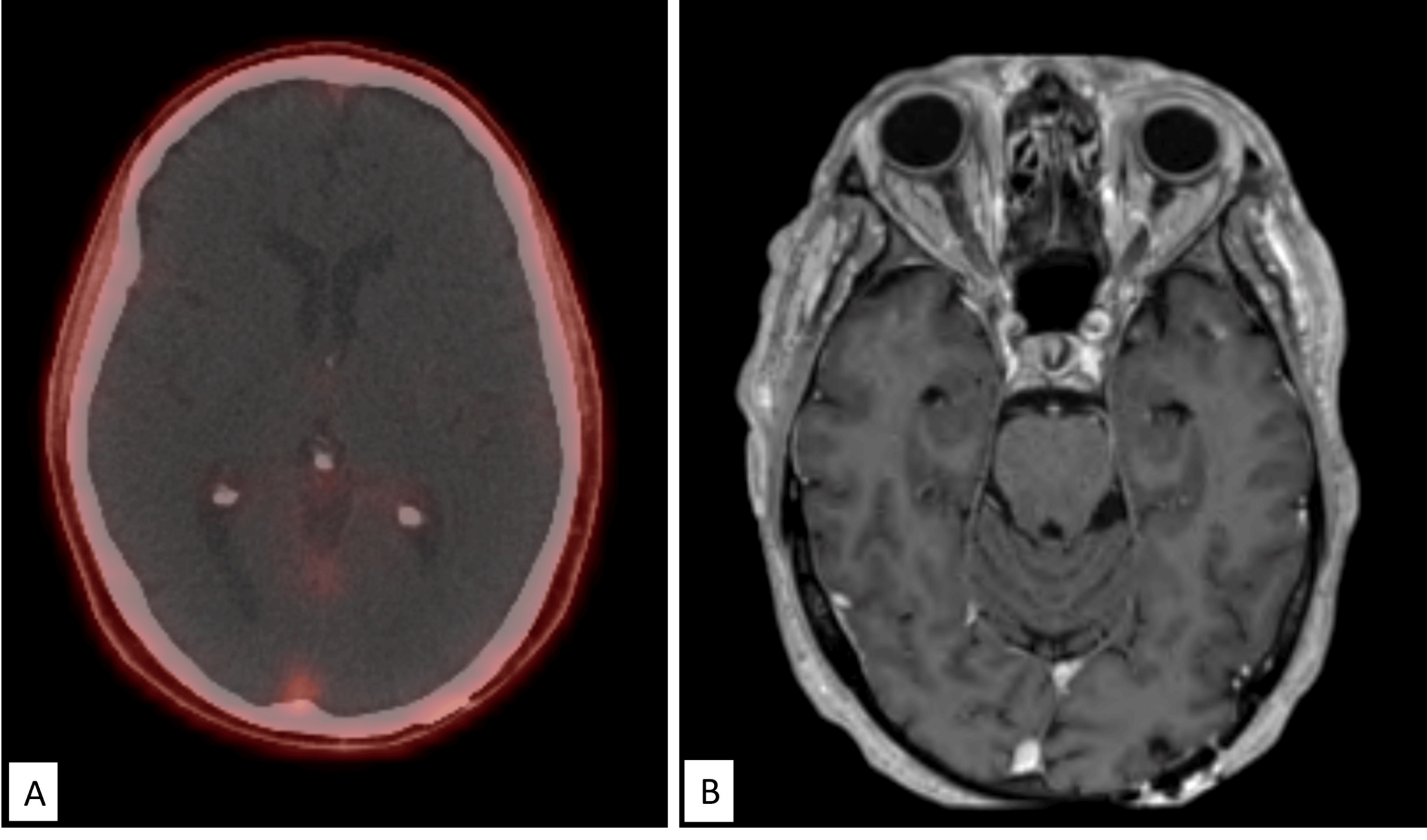
Posttreatment imaging at 15 months following GKRS (A) Complete metabolic resolution of focal PSMA activity in the left occipital lobe peripherally. (B) High-resolution gadolinium double-contrast MRI of the brain showing posttreatment encephalomalacia but no evidence of recurrent or residual malignant disease. GKRS, Gamma Knife radiosurgery; PSMA, prostate-specific membrane antigen

Two weeks after GKRS, the patient was initiated on ADT and apalutamide. The patient’s PSA declined from 5.42 ng/mL two days prior to GKRS to 0.02 ng/mL after 12 months of systemic therapy. Repeat [^68^Ga]PSMA PET/CT and MRI of the brain at 15 months after GKRS revealed stable posttreatment changes, with no new or recurrent tumors (Figure 4).

Caris molecular profiling performed on the resected brain metastatic lesion revealed pathogenic mutations in the *ATM*, *SF3B1*, and *WRN *genes. Taken together, these findings demonstrated durable and effective local and systemic disease control.

## Discussion

Brain metastatic prostate adenocarcinoma without nodal or osseous metastases is a particularly rare occurrence. Current treatment options for these metastases include surgical resection and brain-directed radiotherapy, including stereotactic radiosurgery.

An important focus of clinical management of metastatic prostate cancer has been the identification of genes involved in homologous recombination repair (HRR), in which mutations have been found in up to 20% of castrate-resistant prostate cancer cases [8,9]. The PROFOUND trial, TOPARP-B study, and TAPUR study showed that tumors with alterations in the *BRCA1*, *BRCA2*, or *ATM *genes demonstrated increased sensitivity to poly (ADP-ribose) polymerase (PARP) inhibitors such as olaparib; however, patients with known brain metastases were excluded from the PROFOUND trial [10-12]. Several ongoing trials are examining the efficacy of combining PARP inhibitors with androgen suppressors, such as the PROpel trial, which suggested the use of olaparib in combination with abiraterone as first-line treatment for metastatic castrate-resistant prostate cancer. With a median rPFS of 7.4 months vs. 3.5 months, olaparib plus abiraterone was favored over placebo plus abiraterone (HR: 0.34; 95% CI: 0.25-0.47; p < 0.001) [10].

A 2020 genetic analysis of tumors from 28 patients with brain metastatic prostate cancer revealed that HRR defects are nearly three times more prevalent in brain metastatic prostate cancer than in patients whose metastases did not involve the brain, particularly in the *BRCA2 *and *ATM *genes [8,9]. Interestingly, significant enrichment of mutations in genes such as *NF1*, *RICTOR*, and *YY1AP1 *was found in brain metastases more commonly than those in other locations, particularly copy number loss of *NF1*, raising the possibility that genes such as *NF1 *may facilitate adaptation to the CNS microenvironment [9]. These observations raise clinical implications regarding MEK/ERK inhibitors in patients with *NF1 *mutations and PARP inhibitors in patients with BRCA1/2 alterations [13]. Such interventions may expand therapeutic options against brain metastatic disease and confer improved systemic control in patients with metastatic prostate cancer.

With a detection rate of >90% at PSA levels exceeding 1 ng/mL, [^68^Ga]PSMA PET/CT has been heavily validated for staging prostate cancer and detection of biochemical recurrence [14-17]. However, multiple non-prostate histologies, including CNS tumors such as gliomas and meningiomas, demonstrate avidity for [^68^Ga]PSMA, thus introducing uncertainty regarding the etiology and treatment of these intracranial lesions without pathologic confirmation [18,19]. PSMA expression in the apical cytoplasmic membrane, a marker of prostatic cells, has been shown to demonstrate greater tracer uptake than the cytoplasmic expression often demonstrated by non-prostatic diseases; however, this difference in expression does not offer a clear diagnosis [19,20].

## Conclusions

Cases involving brain metastatic prostate adenocarcinoma without intervening nodal or osseous metastases are particularly rare and require multidisciplinary review for accurate diagnosis and treatment. Combining radiographic findings from [^68^Ga]PSMA PET/CT with immunohistochemical studies targeting biomarkers such as PSA and PSAP, as shown in this case, enables more accurate identification of metastatic prostate carcinomas, compared to other non-prostatic etiologies. Subsequent treatments, surveillance, and long-term management can then be arranged. This case demonstrates that the incorporation of clinical, radiographic, and histopathologic data is essential to make the appropriate diagnosis and potentially guide appropriate multidisciplinary current and future therapeutic options.
